# Targeting mutant BRAF in colorectal cancer

**DOI:** 10.1186/1753-6561-7-S1-O8

**Published:** 2013-01-30

**Authors:** David Carroll, R Carson, S Van Schaeybroeck, PG Johnston

**Affiliations:** 1 Queens University, Belfast, United Kingdom

## Background

Mutations in BRAF V600E oncogene (BRAFMT) occurs in 8-15% of colorectal cancer (CRC) patients1. This mutation constitutively activates MAPK signalling, resulting in a proliferative and survival advantage for the tumour cells and oncogenic BRAF status has been linked with poor prognosis2. Despite introduction of the BRAFMT specific inhibitor Vemurafenib in metastatic melanoma3, there is no effective treatment strategy for BRAFMT CRC patients. This study aimed to assess the effectiveness of Ganetespib (HSP90 inhibitor), the multi-kinase inhibitor (CRAF/VEGFR/PDGFR) Sorafenib and the BRAFMT inhibitor Vemurafenib in BRAFMT CRC cell line models.

## Methods

BRAF MT RKO F6-8 (MT/WT) and isogenic wild-type T29 (null/WT) cell lines were used. MTT assays were used to determine IC50 values. Protein expression was determined by Western Blotting. Levels of apoptotic cells were assessed by flow cytometry.

## Results

The RKO BRAFMT cell line was equally sensitive to Sorafenib and Ganetespib compared to the isogenic BRAFWT clone (IC50 1.4µM vs. 0.9 µM and 0.86 nM vs. 0.79nM respectively). Vemurafenib treatment resulted in a strong decrease in MAPK signalling and showed greater specificity towards BRAFMT cells (IC50 of 2.4µM vs. 3.3 µM) than BRAFWT cells in cell viability assay. Western blotting showed that neither Sorafenib nor Ganetespib had an observable effect in targeting mutant BRAF. Sorafenib did have some effect at targeting downstream MAPK signalling however Ganetespib had no observable effect on targeting the MAPK pathway in BRAF mutant cell lines.. Vemurafenib targeted BRAF and downstream MEK activation in both mutant-type cell lines but not in the wild-type cell lines. We also found that treatment with Vemurafenib induced activation of the STAT3 survival pathway, highlighting a resistance pathway which may be activated in response to treatment in BRAFMT cell lines.

## Conclusions

Despite the number of compounds which can target BRAFMT cell lines through inhibition of mutant BRAF directly or indirectly through inhibition of other pathways, our studies have shown that Vemurafenib is the most effective treatment strategy in the cell line model tested. Effects of Ganetespib or Sorafenib treatment did not display specificity towards BRAFMT cells alone nor an effect on mutant BRAF. Vemurafenib has shown selectivity towards BRAFMT cells in which a reduction in MAPK signaling is achieved along with induction of apoptosis. Evidence of the emergence of a potential resistance mechanism via STAT3 following Vemurafenib treatment was also found giving insight into the kinome reprogramming event which takes place following treatment.
